# Extensive Surgical Emphysema and Pneumomediastinum Following Chest Drain Insertion in a Patient With Bullous Chronic Obstructive Pulmonary Disease (COPD): A Case Report

**DOI:** 10.7759/cureus.96253

**Published:** 2025-11-06

**Authors:** Sindhuja Kannan, Mohd Imran Patel, Riya M Jacob

**Affiliations:** 1 General Medicine, Prince Charles Hospital, Merthyr Tydfyl, GBR; 2 Internal Medicine, Prince Charles Hospital, Merthyr Tydfyl, GBR

**Keywords:** bullous lung disease, chest drain, copd, pneumomediastinum, pneumothorax, : subcutaneous emphysema

## Abstract

Subcutaneous (surgical) emphysema and pneumomediastinum are recognized complications of thoracic procedures. They are often self-limiting but may occasionally indicate serious underlying pathology or precipitate life-threatening deterioration, particularly in patients with chronic obstructive pulmonary disease (COPD) and bullous lung disease. We present the case of an 87-year-old male patient with known COPD who was admitted with worsening dyspnoea and a productive cough. Imaging revealed a large left-sided pneumothorax, and emergency needle decompression followed by intercostal chest drain insertion was performed. Shortly after the procedure, the patient developed extensive surgical emphysema extending from the face to the upper abdominal wall. Computed tomography (CT) of the thorax demonstrated bilateral pneumothoraces, severe pneumomediastinum, and diffuse bullous emphysema. Despite supportive measures and multidisciplinary discussion, the patient’s condition deteriorated, and care was redirected toward comfort measures in line with his wishes. This case highlights a severe complication of chest drain insertion in a patient with bullous COPD, likely precipitated by rupture of pre-existing bullae and air tracking through tissue planes. Awareness of this risk, meticulous procedural technique, and early post-procedure imaging are critical. Even standard thoracic interventions can result in catastrophic complications in patients with fragile lung parenchyma, emphasizing the need for vigilant monitoring and multidisciplinary management.

## Introduction

Subcutaneous emphysema refers to the abnormal presence of air within subcutaneous tissues, often identified clinically by swelling and palpable crepitus. Although typically benign, it can sometimes indicate a serious underlying pathology. Common causes include pneumothorax, pneumomediastinum, or injury to the tracheobronchial tree or oesophagus. Thoracic interventions - particularly chest drain insertion - are recognised iatrogenic causes. Patients with chronic obstructive pulmonary disease (COPD) and bullous lung disease are at increased risk, as fragile alveolar walls predispose to rupture and allow air to dissect along fascial planes into the mediastinum and subcutaneous tissues.

Although subcutaneous emphysema is often self-limiting, severe surgical emphysema following intercostal chest drain insertion in COPD patients is rare (reported in approximately 2%-20 % of cases) and may signal a significant underlying air leak or barotrauma, especially in those with bullous lung disease. This case report describes extensive surgical emphysema and pneumomediastinum following intercostal chest drain insertion in an elderly patient with bullous COPD, illustrating the importance of careful procedural technique, early imaging, and multidisciplinary care.

## Case presentation

An 87-year-old male patient with a history of COPD and osteoarthritis presented to the emergency department on September 4, 2025, with three days of worsening dyspnoea and one day of productive cough with rusty sputum. He was a current smoker (10-15 cigarettes per day) and denied alcohol use.

On arrival, he was acutely dyspnoeic with an oxygen saturation of 62% on room air, heart rate 120 beats/min, blood pressure 140/80 mmHg, and respiratory rate 32 breaths/min. Chest auscultation revealed widespread wheeze with markedly reduced air entry on the left. Formal spirometry or extensive laboratory investigations were not performed, as the patient presented in acute respiratory distress and required immediate stabilisation.

A chest radiograph demonstrated a large left-sided pneumothorax. Emergency needle decompression (14G cannula, second intercostal space, mid-clavicular line) was performed, resulting in immediate improvement in oxygenation. Due to persistent hypoxia on arterial blood gas (partial pressure of oxygen (PaO₂) 7.2 kPa on 60% oxygen; pH 7.31; partial pressure of carbon dioxide (pCO₂) 8.1 kPa), a 28F intercostal chest drain was inserted in the fifth intercostal space (mid-axillary line) using the Seldinger technique under aseptic conditions with -20 cm H₂O suction.

A post-procedure radiograph showed early subcutaneous emphysema around the left chest wall. The patient received supplemental oxygen, intravenous antibiotics, and analgesia. Over the next 24 hours, the subcutaneous emphysema progressed rapidly, involving the chest, neck, face, and upper abdominal wall.

**Figure 1:**
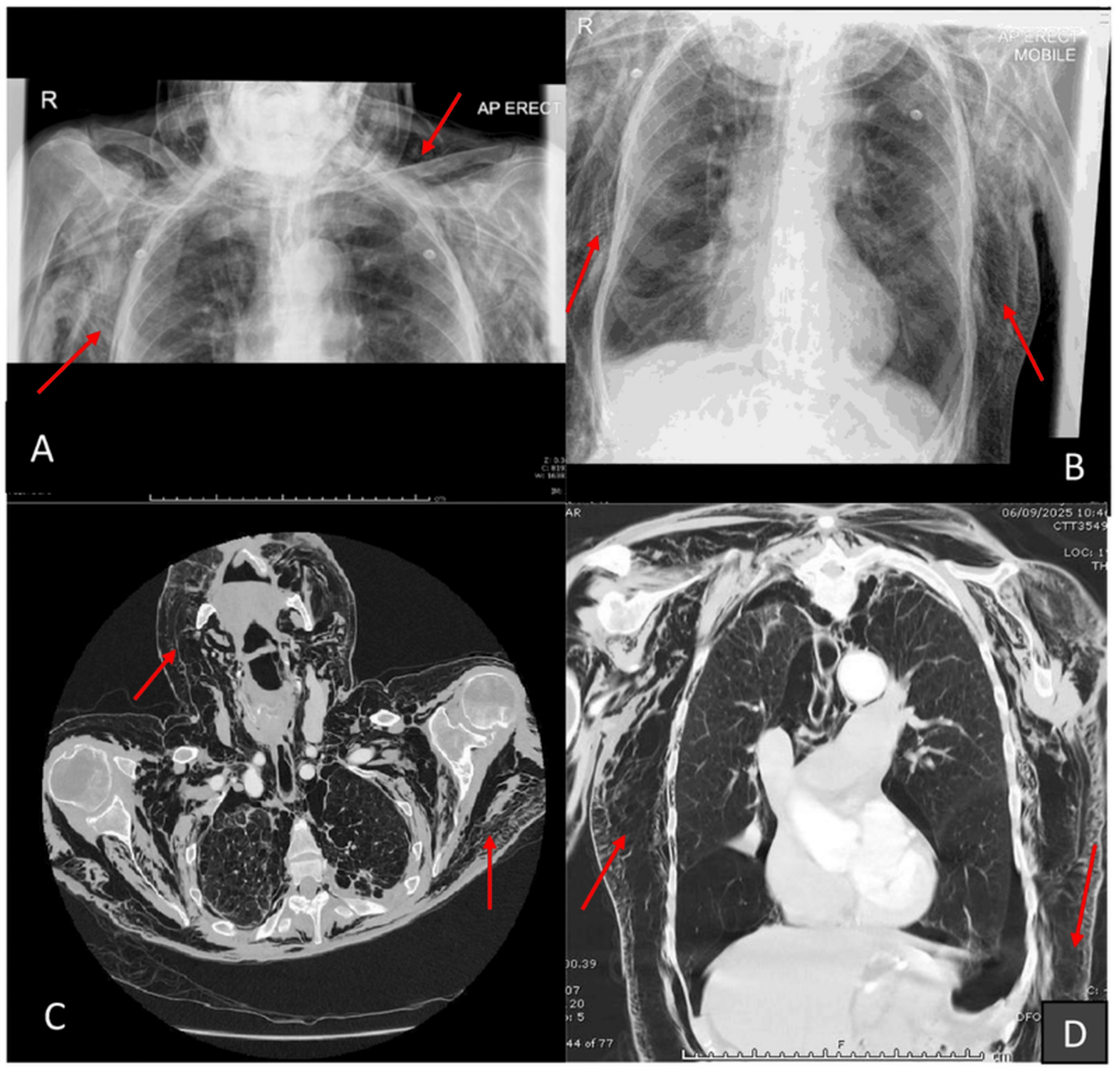
(A, B) Chest radiographs demonstrating extensive surgical emphysema extending into the neck and abdomen (red arrows). (C) Axial CT image showing marked subcutaneous emphysema extending from the chest wall into the neck. (D) Coronal CT image depicting extensive emphysema extending inferiorly into the abdominal region. Red arrows indicate areas of subcutaneous emphysema. Orientation markers and standard lung-window settings were used.

On September 6, 2025, CT of the thorax demonstrated extensive subcutaneous emphysema extending from the face to the upper abdomen, severe pneumomediastinum, bilateral pneumothoraces (both new since initial chest X-ray (CXR)), and diffuse bullous emphysema without evidence of tracheobronchial or oesophageal injury (Figure 1).

The findings and prognosis were discussed with the cardiothoracic and surgical teams, and management options included surgical drains and intensive-care support. In view of advanced COPD, poor prognosis, and the patient’s previously expressed preferences, the decision was made to redirect care toward palliation. The patient died on September 6, 2025.

The chronological progression of the patient’s condition, interventions, and outcomes is summarised in Table 1.

**Table 1:** Clinical Course and Timeline

Date	Event
Sept 1–3, 2025	Worsening shortness of breath and productive cough
Sept 4, 2025	Presented to ED with acute dyspnoea; chest X-ray (CXR): left pneumothorax
Sept 4, 2025	Needle decompression performed; transient improvement
Sept 4, 2025	Seldinger chest drain inserted; surgical emphysema noted post-procedure
Sept 5, 2025	Chest drain removed; progressive surgical emphysema from face to upper abdomen
Sept 6, 2025	CT thorax: extensive subcutaneous emphysema, severe pneumomediastinum, bilateral pneumothoraces, bullous emphysema; no major airway injury
Sept 6, 2025	Discussed with cardiothoracic and general surgery; surgical drains and ICU considered
Sept 6, 2025	Prognosis deemed poor; patient transitioned to comfort care; death later that day

## Discussion

Subcutaneous emphysema most commonly develops when alveolar rupture allows air to travel along perivascular sheaths into the mediastinum and subcutaneous tissues - a mechanism known as the Macklin effect [1,2]. In patients with bullous COPD, fragile alveoli are prone to rupture even with minimal barotrauma, and iatrogenic injury can exacerbate air leaks [3]. Chest-drain insertion may worsen this process through direct pleural or parenchymal trauma, excessive negative suction, or rupture of adjacent bullae [4-6].

CT confirmed the absence of tracheobronchial or oesophageal injury, supporting a parenchymal source for the air leak. Massive surgical emphysema can present dramatically and, in rare cases, lead to airway compromise, respiratory distress, or venous obstruction. Imaging - particularly CT - is critical for defining extent and excluding life-threatening causes [7,8].

Management strategies range from conservative treatment with high-flow oxygen to active decompression using subcutaneous drains, small-bore catheters, or “fish-gill” incisions [9,10]. Prevention strategies include meticulous chest-drain insertion technique, use of ultrasound to identify optimal sites, and avoidance of excessive negative suction.

Similar cases have been reported rarely in the literature [6,9,10], with varying degrees of severity. In patients with advanced COPD and poor physiological reserve, as in this case, escalation must be balanced with patient-centred goals and prognosis.

This case underscores that even routine thoracic procedures can result in catastrophic outcomes in patients with fragile lung parenchyma and emphasises the value of early imaging, multidisciplinary communication, and careful suction management.

## Conclusions

Extensive surgical emphysema and pneumomediastinum are rare but serious complications of chest-drain insertion, particularly in patients with bullous COPD. Awareness of risk factors, careful procedural technique, and early imaging are essential for prompt recognition and appropriate management. In patients with advanced disease and limited physiological reserve, treatment decisions should align with prognosis and patient preferences.

More awareness and simulation-based training in chest-drain insertion may help reduce procedural complications in fragile lungs. This case highlights the importance of multidisciplinary collaboration and vigilant monitoring to prevent, detect, and manage such complications effectively.
